# A Novel TetR Family Transcriptional Regulator, CalR3, Negatively Controls Calcimycin Biosynthesis in *Streptomyces chartreusis* NRRL 3882

**DOI:** 10.3389/fmicb.2017.02371

**Published:** 2017-11-29

**Authors:** Lixia Gou, Tiesheng Han, Xiaoxia Wang, Jingxuan Ge, Wenxiu Liu, Fen Hu, Zhijun Wang

**Affiliations:** ^1^School of Life Sciences, North China University of Science and Technology, Tangshan, China; ^2^Hebei Province Key Laboratory of Occupational Health and Safety for Coal Industry, School of Public Health, North China University of Science and Technology, Tangshan, China; ^3^School of Pharmacy, North China University of Science and Technology, Tangshan, China; ^4^State Key Laboratory of Microbial Metabolism, School of Life Sciences and Biotechnology, Shanghai Jiao Tong University, Shanghai, China

**Keywords:** TetR family regulator, CalR3 ligands, biosynthesis, calcimycin, *Streptomyces chartreusis*

## Abstract

Calcimycin is a unique ionophoric antibiotic that is widely used in biochemical and pharmaceutical applications, but the genetic basis underlying the regulatory mechanisms of calcimycin biosynthesis are unclear. Here, we identified the *calR3* gene, which encodes a novel TetR family transcriptional regulator and exerts a negative effect on calcimycin biosynthesis. Disruption of *calR3* in *Streptomyces chartreusis* NRRL 3882 led to significantly increased calcimycin and its intermediate cezomycin. Gene expression analysis showed that the transcription of *calR3* and its adjacent *calT* gene were dramatically enhanced (30- and 171-fold, respectively) in GLX26 (Δ*calR3*) mutants compared with the wild-type strains. Two CalR3-binding sites within the bidirectional *calR3-calT* promoter region were identified using a DNase I footprinting assay, indicating that CalR3 directly repressed the transcription of its own gene and the *calT* gene. *In vitro* electrophoretic mobility shift assays suggested that both calcimycin and cezomycin can act as CalR3 ligands to induce CalR3 to dissociate from its binding sites. These findings indicate negative feedback for the regulation of CalR3 in calcimycin biosynthesis and suggest that calcimycin production can be improved by manipulating its biosynthetic machinery.

## Introduction

**FIGURE 1:**
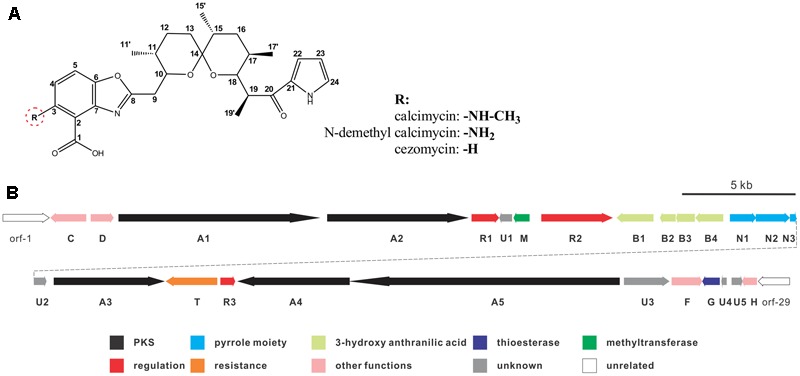
Chemical structure of calcimycin and its precursors **(A)** and genetic organization of the calcimycin gene cluster **(B)**.

Calcimycin, *N*-demethyl calcimycin, cezomycin, CP-61, AC7230, and X-14885A are structurally unique ionophoric antibiotics of the pyrrole polyether family (Boeckman et al., 1991; Wu et al., 2013). Calcimycin, *N*-demethyl calcimycin, and cezomycin are produced by *Streptomyces chartreusis* NRRL 3882 (David and Kergomard, 1982) and feature an α-ketopyrrole, a spiroketal ring, and a benzoxazole, differing only in the substituent group of C-3 on benzoxazole (**Figure 1A**). A well-known prototype of a polyether ionophore, calcimycin has been studied for its underlying mechanism of action and biosynthetic pathway. Calcimycin has shown activity against fungi and Gram-positive bacteria and can inhibit ATPase, uncouple oxidative phosphorylation of mammalian cells, induce the acrosome reaction of mammalian spermatozoa, and induce apoptosis, etc. (Reed, 1976; Tateno et al., 2013; Bloemberg and Quadrilatero, 2016). These diverse effects allow its broad use.

Calcimycin’s biosynthetic pathway is not well understood. The gene cluster for calcimycin biosynthesis has been cloned, and the functions of several structural genes have been elucidated (Wu et al., 2011, 2013); with screens for potential antibiotics, chemical and combinatorial biosynthesis methods have been developed to generate novel calcimycin derivatives (David and Emadzadeh, 1982; Prudhomme et al., 1986; Gou et al., 2013). However, the molecular regulatory mechanisms of pathway-specific regulators for calcimycin production are not clear. From a sequence analysis of the calcimycin biosynthetic gene cluster, three open reading frames (ORFs) have been identified, namely, *calR1, calR2*, and *calR3*, which may have potential regulatory functions, but this has not been reported. CalR1 is a putative TylR family regulator, and CalR2 contains an ATP-binding site at the N-terminus and a DNA-binding motif at the C-terminus. CalR3 contains a helix-turn-helix (HTH) motif that is responsible for DNA binding and it is similar to the TetR family regulators (Cuthbertson and Nodwell, 2013). Thus, studies of putative regulatory genes may suggest how calcimycin is synthesized and allow calcimycin production via its biosynthetic machinery (Routh et al., 2009; Xu et al., 2012).

To address this, we characterized the regulatory roles of CalR3 in calcimycin biosynthesis using disruption, complementation, and transcriptional analysis, and we found that CalR3 acts as a negative regulator in the calcimycin biosynthetic pathway. Calcimycin yield was improved ∼8-fold after the disruption of *calR3*, and *calT* and *calR3* transcription was increased in the Δ*calR3* strain. Finally, CalR3 specifically bound to a core region of a bidirectional promoter between *calT* and *calR3*, blocking the transcription of these two genes. Therefore, we proposed a negative feedback model for the role of *calR3* in the calcimycin biosynthetic pathway.

## Materials and Methods

### Strains, Plasmids, and Cultivation Conditions

The strains and plasmids used in this study are listed in **Table 1**. Cultivation conditions, sporulation, two-parental conjugation and solid fermentation of *S. chartreusis* strains were performed as described previously (Gou et al., 2013). For isolation of chromosomal DNA and RNA, *S. chartreusis* NRRL3882 was grown in liquid TSBY medium at 30°C. For spore collection and two-parental conjugation, *S. chartreusis* NRRL3882 was cultivated at 30°C on solid SFM. TSBY medium was also used for growth curve analysis. Genomic DNA isolation of *S. chartreusis* strains were performed according to Kieser’s group (Kieser et al., 2000). Total RNA was isolated with an RNA isolation kit (Tiangen, China) according to the method of Hopwood’s group (Hopwood et al., 2010). Growth conditions, plasmid isolation, and manipulation of *Escherichia coli* strains were carried out as described by Sambrook and Russell (2001).

**Table 1:** Strains and plasmids used in this study.

Strain or plasmid	Characteristics^1^	Source
***Streptomyces chartreusis***		
NRRL 3882	A23187 production, wild-type	NRRL
GLX26 (Δ*calR3*)	*calR3*-deletion mutant	This work
GLX29 (Δ*calR3+calR3*)	Δ*calR3* complementation strain	This work
***Escherichia coli* strains**		
DH10B	F^-^ *recA lacZ* Δ*M15*	Invitrogen
ET12567(pUZ8002)	Cml, Kan, *dam dcm hsdS* Tra^+^ Cml	Kieser et al., 2000
BW25113/pIJ790	RepA101(ts), araBp-*gam-bet-exo*, AraC, Cml	Kieser et al., 2000
BL21(DE3)/plysE	F^-^ *dcm ompT hsdS* (*rB*^-^ *mB*^-^) *gal*	Stratagene
	(DE3) [pLysE Cml]	
**Plasmids**		
pBluescript II SK(+)	*bla, lacZ, ori^f1^*	Stratagene
pJTU2170	*aac(3)IV, lacz, rep^puc^*, attΦC31, *oriT*	Huang et al., 2011
pET28a(+)	Plasmid for gene expression	Novagen
p16F9	Cml	Wu et al., 2011
pJTU3791	p16F9 derived plasmid carrying a apramycin resistance gene and a defective *calR3*	This work
pJTU3795	pJTU2170 derived plasmid carrying *calR3* for expression in *Streptomyces*	This work
pET28a-calR3	pET28a(+) derived plasmid	This work
	for *calR3* expression	

*^1^Cml, chloramphenicol resistance; Kan, kanamycin resistance; *aac(3)IV*, apramycin resistance.*

### *In Silico* Analysis of *calR3* and *calT*

The complete sequence of the calcimycin biosynthetic gene cluster can be found in GenBank (accession No. HM452329). Similarity and conserved domain analyses were performed using NCBI BlastP (Johnson et al., 2008) and CD-search (Marchler-Bauer et al., 2015), respectively.

### Disruption and Complementation of the *calR3* Gene

The *calR3* gene in *S. chartreusis* was disrupted by inserting the apramycin resistance gene *aac(3)IV* with REDIRECT^R^ technology, as described previously (Gou et al., 2013), and the *aac(3)IV-oriT* cassette was amplified from the pIJ773 plasmid using primers calR3-F1 and calR3-F2 (Supplementary Table 1). The obtained PCR product (1.38 kb) was introduced into BW25113/pKD46 harboring the fosmid p16F9 target against the *calR3* gene, which generated the mutant plasmid pJTU3791 (Δ*calR3*). The introduction of pJTU3791 into the *S. chartreusis* strains by conjugation and selection of double-crossover mutant strains through apramycin resistance were performed according to Kieser’s group (Kieser et al., 2000). Validation of the recombinant strain was performed by PCR analysis using primers calR3-F3 and calR3-F4 (Supplementary Table 1).

For the complementation of strain GLX26(Δ*calR3*), the intact *calR3* gene was amplified from *S. chartreusis* NRRL 3882 genomic DNA with the primers calR3-F5 and calR3-F6 using a high-fidelity DNA polymerase (KOD-plus, TOYOBO). The resulting PCR fragment was cloned into an integrative plasmid, pJTU2170, that was derived from plasmid pIB139 (Huang et al., 2011), resulting in pJTU3795. The *calR3*-complemented strain GLX29 (Δ*calR3:calR3*) was generated by introducing pJTU3795 into GLX26 (Δ*calR3*) via conjugation.

### Detection of Extracellular and Intracellular Calcimycin

The *S. chartreusis* NRRL 3882 and GLX26 strains were inoculated into TSBY media and incubated at 30°C for 72 h, then 2% of the seed cultures were inoculated into the liquid SFM media to incubate for an additional 5 days. The culture broths were filtered using gauze and the residues were washed with 50 ml TSBY medium to remove extracellular calcimycin adhered to the medium particles. The collected filtrate and the mycelia were extracted with ethyl acetate separately. The ethyl acetate was evaporated, redissolved in 1 ml methanol and analyzed using HPLC.

### HPLC Analysis of Metabolites

The *S. chartreusis* NRRL 3882 WT strain, GLX26 (Δ*calR3*), and GLX29 (Δ*calR3:calR3*) were grown on solid SFM medium at 30°C for 7 days. Culture pretreatment and HPLC analysis conditions were performed as described previously (Gou et al., 2013), with a slight modification: the linear gradient elution of methanol was from 65 to 100%.

### RNA Isolation and Gene Transcription Analysis by RT-qPCR

Mycelia of *S. chartreusis* NRRL 3882 and GLX26 strains grown in TSBY medium were collected after 96 h of culturing and individually frozen in liquid nitrogen. Total RNA extractions were performed with a total RNA purification kit (Tiangen, China) following the operating manual. Total RNA samples were digested by DNase I to remove contaminating genomic DNA, and the purity and quantity of the RNA samples were measured using a ScanDrop 200 spectrophotometer and agarose gel electrophoresis. The DNA-free RNA samples (2 μg) were reverse-transcribed with the Fast Quant RT kit (Tiangen, China) according to the manufacturer’s instructions. Transcription analysis of intergenic regions of the *cal* genes was performed to determine polycistrons according to the methods of Xu’s group (Xu et al., 2013). Genomic DNA was used as a template to amplify all regions as positive controls, and DNase I-treated RNA was used as a negative control (primers in Supplementary Table 1). Gene transcriptional analysis was performed by real-time quantitative PCR, with the obtained cDNAs as templates and using the listed primers. The *16S rDNA* gene was used as an internal reference. Experiments were performed using the SuperReal qPCR PreMix (SYBR Green) and analyzed with an ABI7900HT Real-Time PCR System using 384-well plates. Each reaction system (20 μl) contained template cDNA, primer pairs (each 300 nM), and 10 μl SuperReal SYBR Green PreMix. The PCR reaction conditions were the following: 95°C for 15 min followed by 40 cycles of 95°C for 10 s, and 60°C for 30 s.

### Over-Expression and Purification of CalR3

A DNA fragment containing intact *calR3* was amplified using KOD-plus DNA polymerase from *S. chartreusis* NRRL 3882 genomic DNA with primers 28aR3-F1 and 28aR3-F2. The obtained PCR fragment was double-digested using *Nde*I and *Eco*RI and then ligated with linearized vector pET28a(+) (Novagen) at the corresponding restriction sites to generate pET28a-calR3. After sequencing validation, pET28a-calR3 was introduced into *E. coli* BL21(DE3)/plysE (Stratagene) for protein over-expression. Cell growth, induction, and harvest conditions were performed as previously described (Wu et al., 2013). Collected cells were re-suspended in binding buffer (20 mM Tris-Cl, pH 8.0, 300 mM NaCl) and disrupted by ultrasonication. After centrifugation, the supernatant was loaded onto a Ni^2+^-NTA column (Qiagen, Germany) that had been pre-equilibrated with binding buffer. The elution condition was a linear-gradient of imidazole from 0 to 500 mM. The purified protein was desalted by a HisTrap desalination column with buffer C (100 mM Tris-HCl, pH 8.0) and measured using SDS-PAGE. Finally, the purified His_6_-tagged CalR3 protein was stored in stock buffer containing 10% glycerol at -80°C.

### EMSA and DNase I Footprinting Assays

For the electrophoretic mobility shift assay (EMSA), target DNA probes were amplified from *S. chartreusis* NRRL 3882 genomic DNA with the primers shown in Supplementary Table 1 and incubated with purified His_6_-tagged CalR3 protein at 37°C for 30 min in 20μl reaction buffer (20 mM Tris-Cl, 100 mM KCl, 10 mM DTT, pH 8.0). Electrophoretic conditions used by Marchler-Bauer et al. (2015) were applied. Gels were stained in 0.5 μg/ml ethidium bromide and analyzed with a gel electrophoresis imaging system.

The DNase I footprinting assays to determine CalR3 protein-binding sites were performed as described by Zianni’s group (Zianni et al., 2006). The 111-bp T-R3L DNA fragment was PCR amplified with primers P17-F1/P17-F2 and inserted into pGM-T vector (TIANGEN) at the *Eco*RV site, and then the plasmid was PCR-amplified using FAM labeled T7 primer and M13R primer to generate the probe. Probes were quantified using a NanoDrop 2000C after gel-purification. For each reaction volume (40 μl), 500 ng of labeled probes were incubated with various amounts of CalR3 protein at 25°C for 30 min. For partial digestion, about 0.6 units DNase I and 400 nmol CaCl_2_ were added to the reaction mixture. Partial digestion, the electrophoresis conditions and data analysis were performed by the methods reported by Wang et al. (2014).

### 5′ Rapid Amplification of cDNA Ends Analysis for TSP Identification

At 96 h of culturing of *S. chartreusis*, NRRL 3882 was used for total RNA extraction, and then total RNA (2 μg) was reverse-transcribed with 2.5 pmol of the gene-specific primer calTSP1 or calR3SP1 using a 5′-Full RACE kit (TAKARA). The generated cDNAs were recovered from agarose gels prior to addition of a poly(dC) tail at the 3′-end of the cDNA by treatment with terminal deoxynucleotidyl transferase. Tailed cDNA was directly amplified with the poly(dG) anchor primer AAP and a second internal gene-specific primer calTSP2 or calR3SP2. The PCR product was diluted 100-fold and then nest-amplified with an additional round of PCR using an AUAP anchor primer and a nested primer, calTSP3 or calR3SP3. Final PCR products were inserted into the cloning vector pMD18-T followed by sequence validation. The first base after the poly(dG) sequence was considered to be the TSP (Transcriptional Start Point).

### Preparation of Fermentation Supernatant and Cezomycin for EMSAs

*Streptomyces chartreusis* NRRL 3882 fermentation cultures grown in SFM liquid medium for 7 days were used for preparation of fermentation supernatant and purified cezomycin. After centrifugation, the culture supernatants were extracted twice with equal volumes of ethyl acetate. The solvent was then concentrated through rotary evaporation, and residues were redissolved in methanol and subjected to EMSAs. The calcimycin intermediate cezomycin used for EMSAs was isolated from *S. chartreusis* NRRL 3882 fermentation cultures as described previously (Gou et al., 2013).

## Results

### *In Silico* Analysis of *calR3* and *calT*

The *calR3* gene encodes a putative TetR family transcriptional regulator (TFR). BlastP analysis shows that CalR3 displays distinct similarity, especially at the N-terminus, to some TFRs such as MonRII in *S. cinnamonensis* (51% identity), AcrR in *E. coli* (22% identity), TylP and TylQ in *S. fradiae* (22 and 19% identities, respectively), and TetR (19% identity) (Supplementary Figure 1A). Conserved-domain prediction indicates that CalR3 contains a TetR-N-type helix-turn-helix (HTH) DNA binding domain at the N-terminus (Supplementary Figure 1B). There is no conserved domain predicted at the C-terminus, indicating that the induction mechanism for CalR3 is unknown.

### CalR3 Acts as a Repressor of Calcimycin Biosynthesis

**FIGURE 2:**
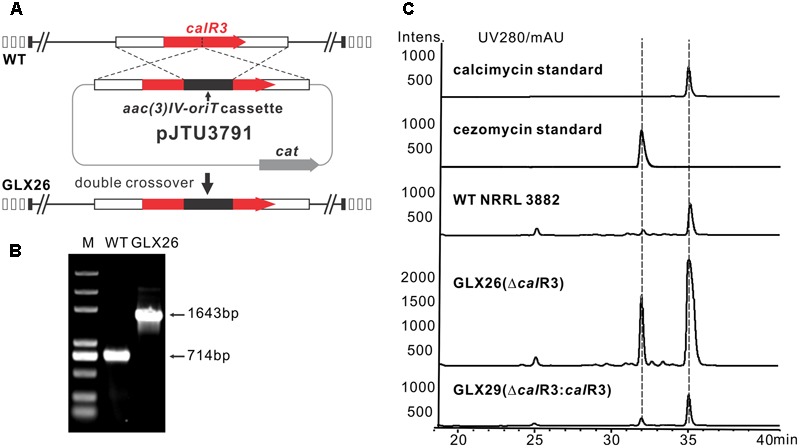
Construction and analysis of *calR3* mutant strain. **(A)** A scheme showing the disruption of *calR3* by the apramycin resistance gene cassette *aac(3)IV* to generate the mutant GLX26. **(B)** The success of construction was confirmed by PCR amplification. For the wild-type gene, the PCR product is 714 bp, while for the mutant, the PCR product is 1,643 bp. **(C)** HPLC traces showing that the inactivation of *calR3* leads to the improvement of calcimycin and cezomycin. The production of calcimycin and cezomycin was restored in the *calR3* complemented strain GLX29.

To elucidate the role of CalR3 in calcimycin biosynthesis, the *calR3* gene was inactivated using a PCR-targeted method (Kieser et al., 2000), and the *calR3* disruption vector pJTU3791 was introduced into *S. chartreusis* NRRL 3882 via conjugation. For inactivation of *calR3* in *S. chartreusis* NRRL 3882, an internal 455-bp DNA sequence in *calR3* was replaced with the *aac(3)IV*-oriT cassette (**Figure 2A**). Candidate mutants with apramycin resistance were confirmed by PCR amplification. A 1,643-bp fragment was produced from the Δ*calR3* mutant strain GLX26 in contrast to a 714-bp DNA fragment from WT (**Figure 2B**). HPLC analysis showed that the calcimycin and cezomycin yields of GLX26 fermentative samples were improved by 8-fold and 30-fold, respectively, compared with WT strains (**Figure 2C**).

To demonstrate that these changes were due to deletion of *calR3*, pJTU3795, which harbors the intact *calR3* gene, was introduced into GLX26. The resulting complemented strain GLX29 (Δ*calR3:calR3*) restored the production of calcimycin and cezomycin (**Figure 2C**). Growth curve analysis of the WT, GLX26, and GLX29 strains in TSBY medium indicated that disruption of *calR3* did not significantly affect cell growth (Supplementary Figure 2). Calcimycin and cezomycin yields from GLX26 were not due to altered cell growth, so CalR3 may function as a negative regulator in the calcimycin biosynthetic pathway.

### Transcription Analysis of *cal* Genes in GLX26 (*ΔcalR3*) and WT Strains

Gene transcription analysis was performed to elucidate the role of CalR3 in calcimycin biosynthesis. The ORFs of *calB2* to *calB4, calN1* to *calN3* and *calA4* to *calA5* were close neighbors (*calB3-B2*: 34 bp; *calB4-B3*: 39 bp; *calN2-N3*: 21 bp; *calA5-A4*: 27 bp) or slightly overlapped (*calN1-N2*: 4 bp overlaps), indicating that *calB4*-*B2, calN1*-*N3* and *calA5-A4* constitute transcriptional units, respectively. Amplifications of intergenic regions between other *cal* genes were performed to identify polycistrons. Data show that all the tested intergenic regions except positive control (*calB4*-*B3*), lacked positive PCR signals, indicating that all *cal* genes, except *calB4*-*B2, calN1*-*N3*, and *calA5-A4*, were located in independent transcriptional units (Supplementary Figure 3).

**FIGURE 3:**
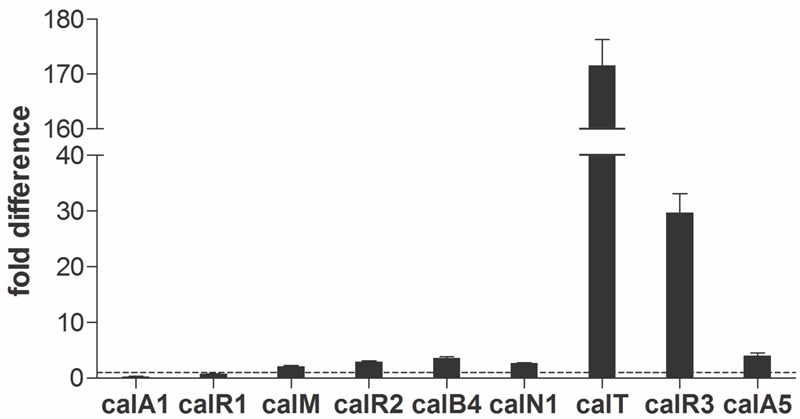
Transcriptional analysis of the *cal* genes. Real-time RT-PCR analysis of *cal* genes transcription levels in GLX-26 and the wild type strain for 96 h incubation.

Real-time PCR was performed with cDNAs of GLX26, and WT strains were cultivated for 96 h (4 days, stationary phase, see Supplementary Figure 2) to investigate the possible effect of *calR3* disruption on the transcription of *cal* genes. The PKS genes *calA1, calA5* (co-transcripts with *calA4*), the benzoxazole gene *calB4* (co-transcripts with *calB3* and *calB2*), the α-ketopyrrole gene *calN1* (co-transcripts with *calN2* and *calN3*), the regulatory genes *calR1, calR2*, and *calR3*, the post-modification gene *calM* (Wu et al., 2013), and the resistance gene *calT* were selected for different locations and putative functions. Real-time PCR analysis showed that the transcription of *calT* and *calR3* were dramatically increased by 171- and 30-fold, respectively, in GLX26 (Δ*calR3*) compared with the WT strain (**Figure 3**).

*calT* encodes a putative antibiotic resistance protein located upstream of *calR3* and is transcribed in the opposite direction (**Figure 1B**). Similar to AcrB, a multidrug efflux protein in *E. coli*, CalT is predicted to harbor two MMPL family domains to alter transmembrane transport (Supplementary Figure 1C). In addition, the gene arrangement of *calR3* and *calT* is almost the same as that of the tetracycline-resistance genes *tetR* and *tetA*; therefore, CalT is hypothesized to export intracellular calcimycin and/or its derivatives out of cells. It intensively hints that CalR3 could regulate the transcription of *calT* and *calR3* and further affect calcimycin and cezomycin yield.

### Identification of the Target Genes of CalR3

**FIGURE 4:**
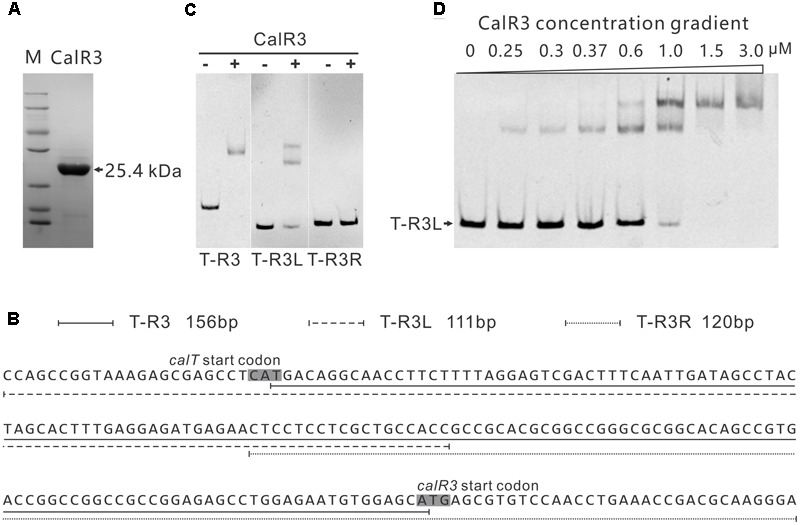
Electrophoretic mobility shift assay (EMSA) analysis of CalR3 protein binding to target promoter regions. **(A)** SDS-PAGE analysis of CalR3, the theoretical molecular mass of His_6_-tagged CalR3 is 25.4 kDa. **(B)** Nucleotide sequences and relative location of probe T-R3, T-R3L, and T-R3R. **(C)** EMSAs of the interaction of probes T-R3, T-R3L, and T-R3R with 0.8 μM of purified His_6_-CalR3 protein. **(D)** EMSAs of the interaction of probe T-R3L with concentration gradient His_6_-CalR3 protein.

To investigate the regulation site of CalR3, EMSAs were performed using DNA fragments containing intergenic regions in the gene cluster as substrates. Soluble full-length recombinant N-terminal His_6_-tagged CalR3 protein with a theoretical molecular weight of 25.4 kDa was over-expressed in *E. coli* and purified (**Figure 4A**). According to transcriptional unit analysis, the 16 intergenic regions from *calC* to *calU4* were amplified using their corresponding primers (Supplementary Table 1). Contiguous genes from *calB4* to *calB2, calN1* to *calN3* and *calA5* to *calA4* were not tested since they are supposed to be co-transcripted, respectively, thus contain no regulation site at DNA level. The results showed that recombinant CalR3 protein had binding activity with the *calT*–*calR3* intergenic region (T–R3) but not for any other of the 15 DNA probes investigated (Supplementary Figure 4 and **Figure 4C**). Because *calT* and *calR3* are divergently transcribed, there may be bidirectional promoters located in the 156-bp intergenic region of T–R3. Thus, we redesigned two fragments: T–R3L and T–R3R (**Figure 4B**). Each fragment covers half of the enlarged T–R3 region and shares 18-bp overlap regions with adjacent fragments. EMSA assays on T–R3L and T–R3R showed that only T–R3L was bound by CalR3 with two retarded bands (**Figure 4B**). EMSA using increasing concentrations of CalR3 for T–R3L confirmed that there were two distinctly shifted bands, and that the T–R3L region might contain two CalR3 binding sites (**Figure 4D**). Different EMSA results between T–R3 and T–R3L may be due to the extended sequence at the 5′-terminus of T–R3L, which could contain a CalR3 recognition site. Precise recognition sites must be known to determine the mechanism of the CalR3 regulation of *calT* and/or *calR3*.

### Determination of the CalR3 Binding Sites

**FIGURE 5:**
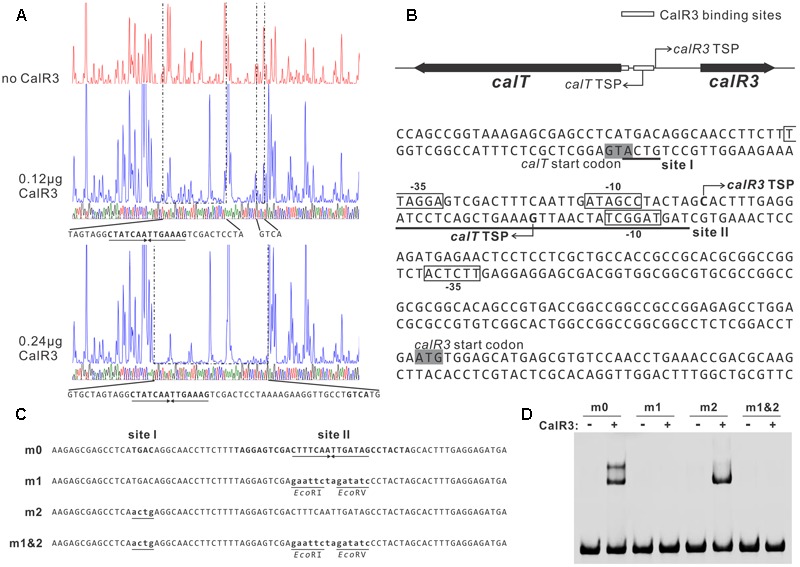
Determination of the binding sites of CalR3 protein. **(A)** DNase I footprinting assay of CalR3 on the T-R3L region. The fluorograms correspond to the control reaction without CalR3 protein and to the protection patterns with increasing CalR3 protein (0.12 and 0.24 μg). **(B)** Nucleotide sequences of the T-R3L promoter region and CalR3-binding site. Solid line, CalR3-binding site; shaded areas, translational start codons; bent arrow, TSPs; boxes, putative –10 and –35 regions. **(C)** Mutations introduced into the CalR3 binding sites I and II regions. Each probe was 80 bp. Probe m0 was WT DNA containing a 14 bp palindromic sequence in site II. Base substitutions were introduced into probe m0 to produce mutated probes m1, m2, and m1&2, respectively. Altered nucleotides are underlined. **(D)** EMSAs with WT DNA probe and mutated probes. Each reaction mixture contained 1.5 μM probe and 0.8 μM of purified His_6_-CalR3 protein.

To illuminate the regulatory sites of CalR3, the 111-bp T–R3L region was PCR-amplified using FAM-labeled primers (P17-F1 and P17-F2, Supplementary Table 1) and analyzed using a DNase I footprinting assay in the presence/absence of His_6_–CalR3. Two protected regions (sites I and II) were identified in the T–R3L region, and the shorter site, I, overlapped with the *calT* translational start codon (**Figures 5A,B**). This explained why T–R3L had two shifted bands, whereas T–R3 had only one site I in T–R3 that was at the leftmost end without any protective bases essential for efficient binding of CalR3. The next reasonable question is the accurate TSPs as well as the relative core promoter regions such as -10 and -35 sites of *calT* and *calR3*, according to which the detailed regulation mechanism of CalR3 would be illuminated.

TetR-family transcriptional regulator proteins typically form symmetric dimers and bind to palindromic sequences (Ramos et al., 2005). Palindromic analysis of the CalR3-binding regions revealed a 14 bp palindromic sequence in the site II region (**Figures 5A,B**). To assess the contributions of site I and the palindromic sequence in the site II region to CalR3 binding, EMSAs were performed with a WT probe m0 or a mutated sequence, as shown in **Figure 5C**, and the molar ratio of probe to CalR3 was properly determined to exhibit two shifted bands on the gel. The affinity of CalR3 for mutated probe m1 which lacked inverted repeats, was abolished completely. For probe m2, base changes were introduced into the site I region and only one retarded band was observed. The affinity of CalR3 for mutated probe m1&2, which was mutated in both inverted repeats and site I, was totally abolished (**Figure 5D**). These findings indicate that the 14 bp palindromic sequence in the site II region is essential for CalR3 binding, and CalR3 binding activity to site I depends on the existence of site II.

### Identification of the TSPs of *calT* and *calR3*

To find the TSPs of *calT* and *calR3*, 5′ RACE was performed using a commercial reverse transcription and cDNA amplification kit. For each gene, 10 clones were sequenced, followed by sequence alignment to determine the TSP site (**Figure 5B** and Supplementary Figure 5). The *calR3* TSP is 104 bp upstream of the *calR3* translational start codon, and the *calR3* TSP is close to the CalR3 binding site II with only a one-base interval; its -10 and -35 regions, as well as the *calT* TSP and its -10 region, were completely covered by the CalR3 binding site II. Thus, CalR3 directly blocks its own transcription and that of the *calT* gene. Additionally, the CalR3 binding site I is located in the *calT* start codon region, indicating tighter regulation for *calT* transcription compared with *calR3*. These data are consistent with the observation that transcription of *calT* and *calR3* was significantly increased after *calR3* was knocked out, and *calT* is increased to a greater extent than *calR3* (**Figure 3**).

### Deletion of *calR3* Enhanced the Export of Calcimycin in GLX26 Strains

Liquid TSBY medium was used to separate the extracellular and intracellular calcimycin. However, the solubility of calcimycin is very low in liquid medium, thus extracellular calcimycin would be trapped together with the medium particles during filtration. Therefore, we washed the mycelia adhere to the gauze to separate extracellular and intracellular calcimycin. HPLC analysis showed that the extracellular and intracellular calcimycin yields of GLX26 were increased 11- and 7-fold, respectively, compared with WT strains (Supplementary Figure 6). The ratio of extracellular to intracellular calcimycin of WT and GLX26 strains were 1:5 and 1:3, respectively, suggesting that disruption of *calR3* enhanced the export of calcimycin.

### Calcimycin and Cezomycin Inhibit the DNA-Binding Activity of CalR3

**FIGURE 6:**
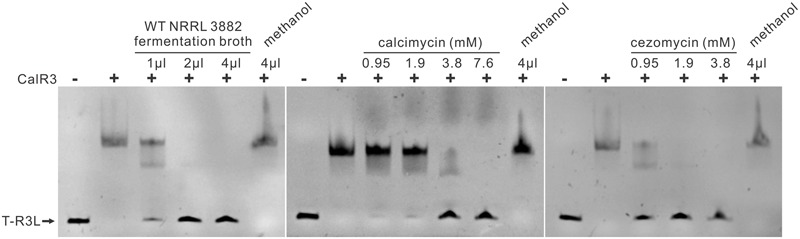
Effects of calcimycin and cezomycin on CalR3 binding to T-R3L region. **(Left)** EMSA of His_6_-tagged CalR3 with concentrated fermentation broth of WT; **(middle)** EMSA of His_6_-tagged CalR3 with calcimycin; **(right)** EMSAs of His_6_-tagged CalR3 with cezomycin. Lanes –, control reaction without protein; lanes +, EMSA reaction in the presence of CalR3 protein. The concentrated fermentation broth, calcimycin and cezomycin were dissolved in methanol, and methanol was used as a solvent control.

Small-molecule compounds act as TFR ligands, typically play roles in the regulation of antibiotic biosynthesis and are related to target regulatory gene(s). Some CalR3 ligands were found to be involved in the regulation of calcimycin biosynthesis. CalT, a putative transmembrane efflux protein, was directly regulated by CalR3; it may pump out the CalR3 ligand(s) during fermentation. To verify this hypothesis, the extracted fermentation supernatant from WT strains was used to evaluate its effects on the affinity of CalR3 for the T–R3L region by EMSA. The results showed that the WT fermentation supernatant inhibited the DNA-binding ability of CalR3 in a concentration-dependent manner (**Figure 6**, left panel), indicating that there were some CalR3 ligands produced by *S. chartreusis* NRRL 3882 in the fermentation broth. EMSAs were performed (**Figure 2C**) using a calcimycin standard, and a purified cezomycin which differs from calcimycin because it lacks an *N*-methyl group on its benzoxazole ring (**Figure 1A**). The data showed that calcimycin-induced dissociation of CalR3 from the T–R3L region (1.9 mM) occurred in a concentration-dependent manner (**Figure 6**, middle panel). Moreover, cezomycin prefers dissociating CalR3 from target DNA regions even at 0.95 mM (**Figure 6**, right panel). Thus, the *N*-methyl group at the C-3 position of calcimycin can suppress the binding of calcimycin to CalR3, but more studies are needed.

## Discussion

Little is known about the regulatory mechanism of calcimycin biosynthesis, but its metabolites can be significantly increased by manipulating regulatory pathways (Chen et al., 2011; Liu et al., 2015). In this study, we characterized a novel TetR-family transcriptional regulator (TFR), CalR3, in *S. chartreusis* NRRL 3882 and demonstrated that it acts as a negative regulator of calcimycin biosynthesis. Other regulators involved in calcimycin production are being characterized in our follow-up work to elucidate the regulatory network of calcimycin biosynthesis.

TetR-family transcriptional regulator genes are usually oriented divergently from their neighboring genes, and most TetR family proteins can tightly control the transcription of both genes by binding to their intergenic region (Ramos et al., 2005; Cuthbertson and Nodwell, 2013). The situation for *calR3* and *calT* is similar. TetR family proteins are the third most common transcriptional regulators and are extensively distributed among bacteria; they harbor an N-terminal HTH DNA-binding motifs and C-terminal domains that interact with ligands (Cuthbertson et al., 2013). However, most TetR family regulators have not been characterized, and the specific function regulated by TFR members has only been reviewed for 481 members of the 354,557 members in SWISS-PROT and TrEMBL (as of June 2017). The TetR family is named based on the well-characterized TetR protein from the Tn10 transposon, which binds to the intergenic region of *tetR* and *tetA*, thus negatively controlling their transcription (Yang et al., 1976). We found that CalR3 used a similar mechanism to directly repress transcription of *calT* and itself by binding to two sites—site I is a 4 bp sequence, GTCA, that overlaps with the *calT* translational start codon, and site II is a 31-bp sequence containing a 14 bp palindromic sequence, TAGGAGTCGACTTTCAATTGATAGCCTACTA, covering the core regions of the *calR3* and *calT* promoters. Similar 4 bp sequences exist in other promoter regions, but CalR3 did not bind to any of these putative sites. CalR3 binding activity to site I was abolished when palindromic sequence in site II was mutated, suggesting the CalR3 binding activity to site I depends on the existence of site II. The two binding sites in the *calT* 5′-UTR region offer more stringent control of *calT* transcription than *calR3*, which was verified by gene transcriptional analysis (**Figure 3**).

Small-molecule compounds have been reported to act as TFR ligands and can allosterically inactivate the DNA-binding activity of TFRs. For example, in the actinorhodin biosynthetic pathway of *S. coelicolor*, the DNA-binding activity of TFR ActR on the efflux pump gene, *actAB*, is blocked by actinorhodin and its 3-ring biosynthetic intermediates (Tahlan et al., 2007). Similarly, avermectin B1 biosynthetic precursor C-5-O-B1 impairs the binding activity of TFR, AveT (Liu et al., 2015). TylP shows 22% identity with CalR3, and the autoregulation of TylP is relieved by γ-butyrolactone (Bignell et al., 2007). In this study, CalR3 is unbound from its target binding sites after interacting with end-product calcimycin and the late pathway intermediate cezomycin. The structure of cezomycin lacks only one *N*-methyl group at the benzoxazole ring compared with calcimycin. However, cezomycin apparently has a preference for dissociating CalR3 from its target DNA regions. Therefore, the *N*-methyl group may offer steric hindrance to interfere with CalR3 with its ligand(s). Consequently, a structural biological study of CalR3 is needed to illuminate the accurate allosteric mechanism and identify the best paired ligand. On the other hand, *calR3* and a suitable ligand provide a novel gene circuit module that can be applied in synthetic biology research such as tetR and aTc (anhydroTetracycline) (Degenkolb et al., 1991; Schubert et al., 2004).

**FIGURE 7:**
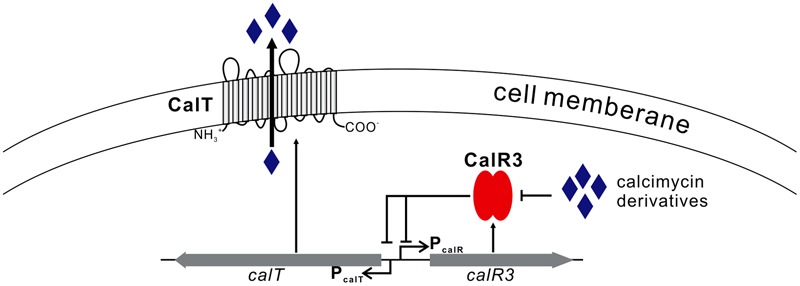
Proposed model of CalR3-mediated regulation of calcimycin production in *Streptomyces chartreusis* NRRL 3882 Black arrows, activation; black bars, repression.

We focused on only one precursor, so other intermediates in the calcimycin biosynthetic pathway, such as *N*-demethyl calcimycin, may also bind to CalR3. More studies are necessary to identify other ligand(s) of CalR3 to better characterize the regulatory mechanisms of calcimycin biosynthesis. The *calT* gene encodes a putative transmembrane efflux pump protein of the MMPL family and can be repressed directly by CalR3. Bacteria and fungi often produce antibiotic metabolites to antagonize other microorganisms. To avoid self-inhibition, the antibiotic metabolite biosynthetic clusters often employ one or more transmembrane export apparatus which is responsible for expelling toxic by-products (Tahlan et al., 2007). The *actAB* operon encodes two export pumps and is involved in driving efficient actinorhodin production and preventing toxic accumulation of antibiotics in the cytoplasm. Pump genes are repressed by the TetR family protein ActR, which has 18% identity with CalR3 (Xu et al., 2012). AcrB, the CalT homolog protein is also a major multidrug exporter in *E. coli* (Murakami et al., 2002). We suggest that background expression of *calR3* in the early stages of *S. chartreusis* growth allows its corresponding protein to directly bind to the bidirectional promoter between *calT* and *calR3* to repress their transcription. When accumulated, calcimycin and its intermediate cezomycin reach a threshold and bind with CalR3 to induce an allosteric effect to prevent CalR3 from binding to DNA. This activates the transcription of both *calR3* and *calT* again (**Figure 7**). Meanwhile, CalT may be involved in expulsion of calcimycin and some intermediates accompanying calcimycin biosynthesis. As *calT* expression increases, excessive calcimycin and cezomycin are removed to ensure an appropriate concentration. Disruption of *calR3* enhanced the accumulation of extracellular calcimycin and improved the ratio of extracellular to intracellular calcimycin yield. This data supports the hypothesis that CalT exports calcimycin and reduces the intracellular inhibition of calcimycin biosynthesis.

This is the first report regarding the regulatory mechanism of calcimycin biosynthesis, and we identified CalR3 as a novel negative regulator targeting *calR3* and *calT* in the calcimycin biosynthetic pathway. Our strategy for enhanced calcimycin yield by engineering of CalR3 and its target gene(s) may be applied to other industrially and commercially important *Streptomyces* strains that have CalR3 homologs to increase antibiotic production.

## Author Contributions

TH, ZW, LG, and XW: conceived and designed the research. LG, XW, JG, WL, and FH: performed the experiments. LG and FH: wrote the manuscript. TH and ZW: revised and approved the final manuscript.

## Conflict of Interest Statement

The authors declare that the research was conducted in the absence of any commercial or financial relationships that could be construed as a potential conflict of interest.

## References

[B1] Bignell D. R. Bate N. Cundliffe E. (2007). Regulation of tylosin production: role of a TylP-interactive ligand. *Mol. Microbiol.* 63 838–847. 10.1111/j.1365-2958.2006.05541.x 17181783

[B2] Bloemberg D. Quadrilatero J. (2016). Caspase activity and apoptotic signaling in proliferating C2C12 cells following cisplatin or A23187 exposure. *Data Brief* 7 1024–1030. 10.1016/j.dib.2016.03.032 27104214 PMC4826590

[B3] Boeckman R. K. Jr. Charette A. B. Asberom T. Johnston B. H. (1991). The chemistry of cyclic vinyl ethers. 6. Total synthesis of polyether ionophore antibiotics of the calcimycin (A-23 187) class. *J. Am. Chem. Soc.* 113 5337–5353. 10.1021/ja00014a029

[B4] Chen Y. Yin M. Horsman G. P. Shen B. (2011). Improvement of the enediyne antitumor antibiotic C-1027 production by manipulating its biosynthetic pathway regulation in *Streptomyces globisporus*. *J. Nat. Prod.* 74 420–424. 10.1021/np100825y 21250756 PMC3064734

[B5] Cuthbertson L. Ahn S. K. Nodwell J. (2013). Deglycosylation as a mechanism of inducible antibiotic resistance revealed using a global relational tree for one-component regulators. *Chem. Biol.* 20 232–240. 10.1016/j.chembiol.2012.11.011 23438752

[B6] Cuthbertson L. Nodwell J. R. (2013). The TetR family of regulators. *Microbiol. Mol. Biol. Rev.* 77 440–475. 10.1128/MMBR.00018-13 24006471 PMC3811609

[B7] David L. Emadzadeh S. (1982). Biosynthesis of the ionophorous antibiotic A23187. *J. Antibiot.* 35 1616–1617. 10.7164/antibiotics.35.1616 6819282

[B8] David L. Kergomard A. (1982). Production by controlled biosynthesis of a novel ionophore antibiotic, cezomycin (demethylamino A23187). *J. Antibiot.* 35 1409–1411. 10.7164/antibiotics.35.1409 6816779

[B9] Degenkolb J. Takahashi M. Ellestad G. A. Hillen W. (1991). Structural requirements of tetracycline-Tet repressor interaction: determination of equilibrium binding constants for tetracycline analogs with the Tet repressor. *Antimicrob. Agents Chemother.* 35 1591–1595. 10.1128/AAC.35.8.1591 1929330 PMC245224

[B10] Gou L. Wu Q. Lin S. Li X. Liang J. Zhou X. (2013). Mutasynthesis of pyrrole spiroketal compound using calcimycin 3-hydroxy anthranilic acid biosynthetic mutant. *Appl. Microbiol. Biotechnol.* 97 8183–8191. 10.1007/s00253-013-4882-1 23666477

[B11] Hopwood D. A. Bibb M. J. Chater K. F. Kieser T. Bruton C. J. Kieser H. M. (2010). Genetic manipulation of *Streptomyces*; a laboratory manual. *J. Cell Biol.* 56 388–399.

[B12] Huang T. Wang Y. Yin J. Du Y. Tao M. Xu J. (2011). Identification and characterization of the pyridomycin biosynthetic gene cluster of *Streptomyces pyridomyceticus* NRRL B-2517. *J. Biol. Chem.* 286 20648–20657. 10.1074/jbc.M110.180000 21454714 PMC3121499

[B13] Johnson M. Zaretskaya I. Raytselis Y. Merezhuk Y. Mcginnis S. Madden T. L. (2008). NCBI BLAST: a better web interface. *Nucleic Acids Res.* 36 W5–W9. 10.1093/nar/gkn201 18440982 PMC2447716

[B14] Kieser T. Bibb M. J. Buttner M. J. Chater K. F. Hopwood D. A. (2000). *Practical Streptomyces Genetics.* Norwich: The John Innes Foundation.

[B15] Liu W. Zhang Q. Guo J. Chen Z. Li J. Wen Y. (2015). Increasing avermectin production in *Streptomyces avermitilis* by manipulating the expression of a novel TetR-family regulator and its target gene product. *Appl. Environ. Microbiol.* 81 5157–5173. 10.1128/AEM.00868-15 26002902 PMC4495205

[B16] Marchler-Bauer A. Derbyshire M. K. Gonzales N. R. Lu S. Chitsaz F. Geer L. Y. (2015). CDD: NCBI’s conserved domain database. *Nucleic Acids Res.* 43 D222–D226. 10.1093/nar/gku1221 25414356 PMC4383992

[B17] Murakami S. Nakashima R. Yamashita E. Yamaguchi A. (2002). Crystal structure of bacterial multidrug efflux transporter AcrB. *Nature* 419 587–593. 10.1038/nature01050 12374972

[B18] Prudhomme M. Guyot J. Jeminet G. (1986). Semi-synthesis of A23187 (calcimycin) analogs. IV. Cation carrier properties in mitochondria of analogs with modified benzoxazole rings. Antimicrobial activity. *J. Antibiot.* 39 934–937. 10.7164/antibiotics.39.934 3093432

[B19] Ramos J. L. Martinez-Bueno M. Molina-Henares A. J. Teran W. Watanabe K. Zhang X. (2005). The TetR family of transcriptional repressors. *Microbiol. Mol. Biol. Rev.* 69 326–356. 10.1128/MMBR.69.2.326-356.2005 15944459 PMC1197418

[B20] Reed P. W. (1976). Effects of divalent cation ionophore A23187 on potassium permeability of rat erythrocytes. *J. Biol. Chem.* 251 3489–3494. 6455

[B21] Routh M. D. Su C. C. Zhang Q. Yu E. W. (2009). Structures of AcrR and CmeR: insight into the mechanisms of transcriptional repression and multi-drug recognition in the TetR family of regulators. *Biochim. Biophys. Acta* 1794 844–851. 10.1016/j.bbapap.2008.12.001 19130905 PMC2729549

[B22] Sambrook J. And Russell D. W. (2001). *Molecular Cloning: A Laboratory Manual* 3rd Edn. New York, NY: Cold Spring Harbor Laboratory.

[B23] Schubert P. Pfleiderer K. Hillen W. (2004). Tet repressor residues indirectly recognizing anhydrotetracycline. *Eur. J. Biochem.* 271 2144–2152. 10.1111/j.1432-1033.2004.04130.x 15153105

[B24] Tahlan K. Ahn S. K. Sing A. Bodnaruk T. D. Willems A. R. Davidson A. R. (2007). Initiation of actinorhodin export in *Streptomyces coelicolor*. *Mol. Microbiol.* 63 951–961. 10.1111/j.1365-2958.2006.05559.x 17338074

[B25] Tateno H. Krapf D. Hino T. Sánchez-Cárdenas C. Darszon A. Yanagimachi R. (2013). Ca^2+^ ionophore A23187 can make mouse spermatozoa capable of fertilizing in vitro without activation of cAMP-dependent phosphorylation pathways. *Proc. Natl. Acad. Sci. U.S.A.* 110 18543–18548. 10.1073/pnas.1317113110 24128762 PMC3831971

[B26] Wang L. Tang H. Yu H. Yao Y. Xu P. (2014). An unusual repressor controls the expression of a crucial nicotine-degrading gene cluster in *Pseudomonas putida* S16. *Mol. Microbiol.* 91 1252–1269. 10.1111/mmi.12533 24471758

[B27] Wu Q. Gou L. Lin S. Liang J. Yin J. Zhou X. (2013). Characterization of the N-methyltransferase CalM involved in calcimycin biosynthesis by *Streptomyces chartreusis* NRRL 3882. *Biochimie* 95 1487–1493. 10.1016/j.biochi.2013.03.014 23583975

[B28] Wu Q. Liang J. Lin S. Zhou X. Bai L. Deng Z. (2011). Characterization of the biosynthesis gene cluster for the pyrrole polyether antibiotic calcimycin (A23187) in *Streptomyces chartreusis* NRRL 3882. *Antimicrob. Agents Chemother.* 55 974–982. 10.1128/AAC.01130-10 21173184 PMC3067094

[B29] Xu D. Liu G. Cheng L. Lu X. Chen W. Deng Z. (2013). Identification of Mur34 as the novel negative regulator responsible for the biosynthesis of muraymycin in *Streptomyces* sp. NRRL30471. *PLOS ONE* 8:e76068. 10.1371/journal.pone.0076068 24143177 PMC3797123

[B30] Xu Y. Willems A. Au-Yeung C. Tahlan K. Nodwell J. R. (2012). A two-step mechanism for the activation of actinorhodin export and resistance in *Streptomyces coelicolor*. *mBio* 3:e00191-12. 10.1128/mBio.00191-12 23073761 PMC3482498

[B31] Yang H. L. Zubay G. Levy S. B. (1976). Synthesis of an R plasmid protein associated with tetracycline resistance is negatively regulated. *Proc. Natl. Acad. Sci. U.S.A.* 73 1509–1512. 10.1073/pnas.73.5.1509 775491 PMC430326

[B32] Zianni M. Tessanne K. Merighi M. Laguna R. Tabita F. R. (2006). Identification of the DNA bases of a DNase I footprint by the use of dye primer sequencing on an automated capillary DNA analysis instrument. *J. Biomol. Tech.* 17 103–113. 16741237 PMC2291779

