# Supplementary material for: A Novel TetR Family Transcriptional Regulator, CalR3, Negatively Controls Calcimycin Biosynthesis in Streptomyces chartreusis NRRL 3882
Source: Front Microbiol. 2017 Nov 29;8:2371. doi: 10.3389/fmicb.2017.02371 (PMC5712553; doi:10.3389/fmicb.2017.02371)
Supplement: Supplementary file 1 [file Data_Sheet_1.PDF]

Supplementary Material

A Novel TetR Family Transcriptional Regulator, CalR3, Negatively Controls Calcimycin Biosynthesis in *Streptomyces chartreusis* NRRL 3882

Lixia Gou[1], Tiesheng Han[1*], XiaoxiaWang[1], Jingxuan Ge[1], WenxiuLiu[1], Fen Hu[1]and ZhijunWang[2*]

1, School of Life Science, Hebei Province Key Laboratory of Occupational Health and Safety for Coal Industry, School of Public Health, School of Pharmacy, North China University of Science and Technology, Tangshan, Hebei, China

2, State Key Laboratory of Microbial Metabolism, School of Life Sciences & Biotechnology, Shanghai Jiao Tong University, Shanghai, China

***Correspondence:**

Tiesheng Han
ts_han@163.com

Zhijun Wang
wangzhijun@sjtu.edu.cn

1 Supplementary Figures and Tables

1.1 Supplementary Table

Supplementary Table 1. Primers used in this study.

Primer	5’ Sequence 3’	Usage
**Gene disruption, complementation, and expression**		
calR3-F1	gaccagggctacgaggcgacgacgatcgagcagatcgccATT CCGGGGATCCGTCGACC	Disruption of *calR3* by REDIRECT[R] Technology
calR3-F2	ggcagcggggtccagctccggctggttggcccagcgcag TGTAGGCTGGAGCTGCTTC	
calR3-F3	TCCAACCTGAAACCGACGCAAGG	Confirmation of *calR3* disruption in GLX26(*ΔcalR3*)
calR3-F4	CCGAAGCGAAGCGAAGACCC	
calR3-F5	GGAATTCCATATGACCGTCGAGAGCCACAC	Complementation of GLX26(*ΔcalR3*)
calR3-F6	CCGGAATTCCACGTGCCAACCCGCACCGG	
28aR3-F1	GGAATTCCATATGAGCGTGTCCAACCTGA	Over expression of His_6-tagged CalR3 protein in *E. coli*
28aR3-F2	CCGGAATTCGAAGCGAAGCGAAGACCC	
**Polycistron determination**		
B3/B4-F1	GAATGCCGTACTCGGCGACCTCC	*calB3-calB4* intergenic region
B3/B4-F2	CGGTCCTCAGGGAGATCAAGGAG	
D/A1-F1	GCGGGGAACGTCGGAAGC	*calD-calA1* intergenic region
D/A1-F2	CGGCTATCGCCGTAGTGAAATCT	
A1/A2-F1	CTCAACAAGGCGACGGGTCT	*calA1-calA2* intergenic region
A1/A2-F2	GCCTCCCACGACACCTCCA	
A2/R1-F1	CCTGCTGCCCCGACTCAC	*calA2-calR1* intergenic region
A2/R1-F2	GATGTTGCGGATGGACTTGC	
U1/M-F1	GGTTGGGCTCGGTGGTCA	*calU1-calM* intergenic region
U1/M-F2	CGGCAGGCTGTTCATAGGGTT	
B1/B2-F1	GCACGACGAACGTCCAGTAGG	*calB1-calB2* intergenic region
B1/B2-F2	GCTGATCATTTGCGGGGTCTA	
N3/U2-F1	AGGGCGAACTGGAGGAGACC	*calN3-calU2* intergenic region
N3/U2-F2	TGGCAGTCCTGGGTGTCGTA	
U2/A3-F1	TCGGGGTCCGCACCAATA	*calU2-calA3* intergenic region
U2/A3-F2	CGCCTCCCAGGTCGTCTCC	
U3/F-F1	GGTGGTACACGCCGCTCAA	*calU3-calF* intergenic region
U3/F –F2	CGTCCTGACTGGTCGAACCC	
**EMSA**		
P1-F1	CATGCTGTCACGCTTCCA	Probe *calC-D* intergenic region
P1-F2	GCATGCGCCCAGAGTG	
P2-F1	CCTGCGATAGGGGTGC	Probe *calD-A1* intergenic region

P2-F2	GGTCGGGTCGTCATGT	
P3-F1	GCGAGTAATTCACGATGAAT	Probe *calA1-A2* intergenic region
P3-F2	ATCGCCAACGCCTCAC	
P4-F1	AGCCACTGGAAACGGGTCC	Probe *calA2-R1* intergenic region
P4-F2	TGTTTCCCCATCTCGGCC	
P5-F1	TCAGGGATCATGACACCGCA	Probe *calU1-M* intergenic region
P5-F2	CGCGCGGGCTCCTGAT	
P6-F1	CTTCTTGTCCGGTCCG	Probe *calM-R2* intergenic region
P6-F2	ATACATCCCCGTCCC	
P7-F1	GGCGAGCAACGGCGTT	Probe *calB1-B2* intergenic region
P7-F2	ACAGTGAGCGGCCGGC	
P8-F1	CCATGCGGAGTTCTCCCT	Probe *calB4-N1* intergenic region
P8-F2	CCATAGCAGTTAAGGGCC	
P9-F1	GAGCTCCAGCACTCGGCG	Probe *calN3-U2* intergenic region
P9-F2	CGCGTCGCTGGCGATG	
P10-F1	ACAGCGAGATGCCCGAC	Probe *calU2-A3* intergenic region
P10-F2	TGGCCGCCGCCTCGAC	
P11-F1	TGACAGGCAACCTTCTT	Probe *calT-R3* intergenic region
P11-F2	TGCTCCACATTCTCCA	
P12-F1	TCCCGATCCTCGGCAGGGA	Probe *calA5-U3* intergenic region
P12-F2	TGCGAACGCTCCGTCCCG	
P13-F1	CGGGGTGATCGTGTGA	Probe *calU3-F* intergenic region
P13-F2	CATGGCCTGGAGCCAC	
P14-F1	TATCCACGGATCGGTG	Probe *calG-U4* intergenic region
P14-F2	CCGACCTCAGAATGGG	
P15-F1	TGCGGGGAACTCCTCT	Probe *calU4-U5* intergenic region
P15-F2	TGGGATCCCTCCAGGT	
P16-F1	ACAGGGGTGTCCTTCCGTG	Probe *calH-orf29* intergenic region
P16-F2	CACCTGTTGGCGTCGG	
P17-F1	CCAGCCGGTAAAGAGCGAGC	Probe T-R3L intergenic region
P17-F2	GGTGGCAGCGAGGAGGAGTT	
P18-F1	CTCCTCCTCGCTGCCACC	Probe T-R3R intergenic region
P18-F2	TCCCTTGCGTCGGTTTCA	
**5' RACE**		
calTSP1	GGATGGAGAGCGAGTC	Identification of TSP of c*alT*
calTSP2	GTGGGCCTGGACAGCAGC	
calTSP3	ACGGCAGCCACCCAGAGT	
calR3SP1	TCGCCTCGTAGCCCTGGTCC	Identification of TSP of c*alR3*
calR3SP2	CCGGGTCTTGGCCTTCTTGC	
calR3SP3	TGCGTCGGTTTCAGGTTGGA	
anchor AAP	GGCCACGCGTCGACTAGTACGGGIIGGGIIGGGIIG	

anchor AUAP	GGCCACGCGTCGACTAGTAC	
**Real-time RT-PCR**		
RTA1-F1	ACCCGACCGCCAGGAGATT	*calA1* internal region
RTA1-F2	CAGCAGACGCCCGAAGGAG	
RTR1-F1	TGGACGAGGTGCTGCACGAT	*calR1* internal region
RTR1-F2	TGGATCAGCCCGTGGAGGTA	
RTM-F1	ACCCTATGAACAGCCTGCCG	*calM* internal region
RTM-F2	CGTCTACACCAAGGATCTGGAGC	
RTR2-F1	GTTCTCCTCCCTCAGCGAACTG	*calR2* internal region
RTR2-F2	AGTACCGCAGCCGCCGTA	
RTB4-F1	CGTCGTGTACTTCCTGCCTACCT	*calB4* internal region
RTB4-F2	TCCAGTGCGCCGTTCGTG	
RTN1-F1	CGTTCACCGACTGCCACGT	*calN1* internal region
RTN1-F2	GCTCCATCAACCCGAGGTAGAC	
RTU2-F1	GACGCGGGCAAGATCAACAC	*calU2* internal region
RTU2-F2	CCCGAGAAGGAAGTCCAGGT	
RTA3-F1	ACACCCAGTGCACCTCCTCGAT	*calA3* internal region
RTA3-F2	TCGGCAACGACATGACGCAG	
RTR3-F1	CGAGCATCCGGGGAAGATCA	*calR3* internal region
RTR3-F2	TCGATCGTCGTCGCCTCGTA	
RTA5-F1	TGTCGGGGAAGTCGTATGTGC	*calA5* internal region
RTA5-F2	ATCGCCAACGCCTCACGC	
RTU3-F1	CAGTCCCGCCATCTGCTGAT	*calU3* internal region
RTU3-F2	GCTTCGCCCAGTCGTACAGA	
RTG-F1	GTGCGTGGTCGACGAGGAAGAAGT	*calG* internal region
RTG-F2	AAGGCGAGCCATGACGAGGTAC	
**DNase I footprinting assay**		
FAM-T7	TAATACGACTCACTATAGGG	*Amplifying calT-calR3* intergenic region
M13R	CAGGAAACAGCTATGACC	

1.2 Supplementary Figures

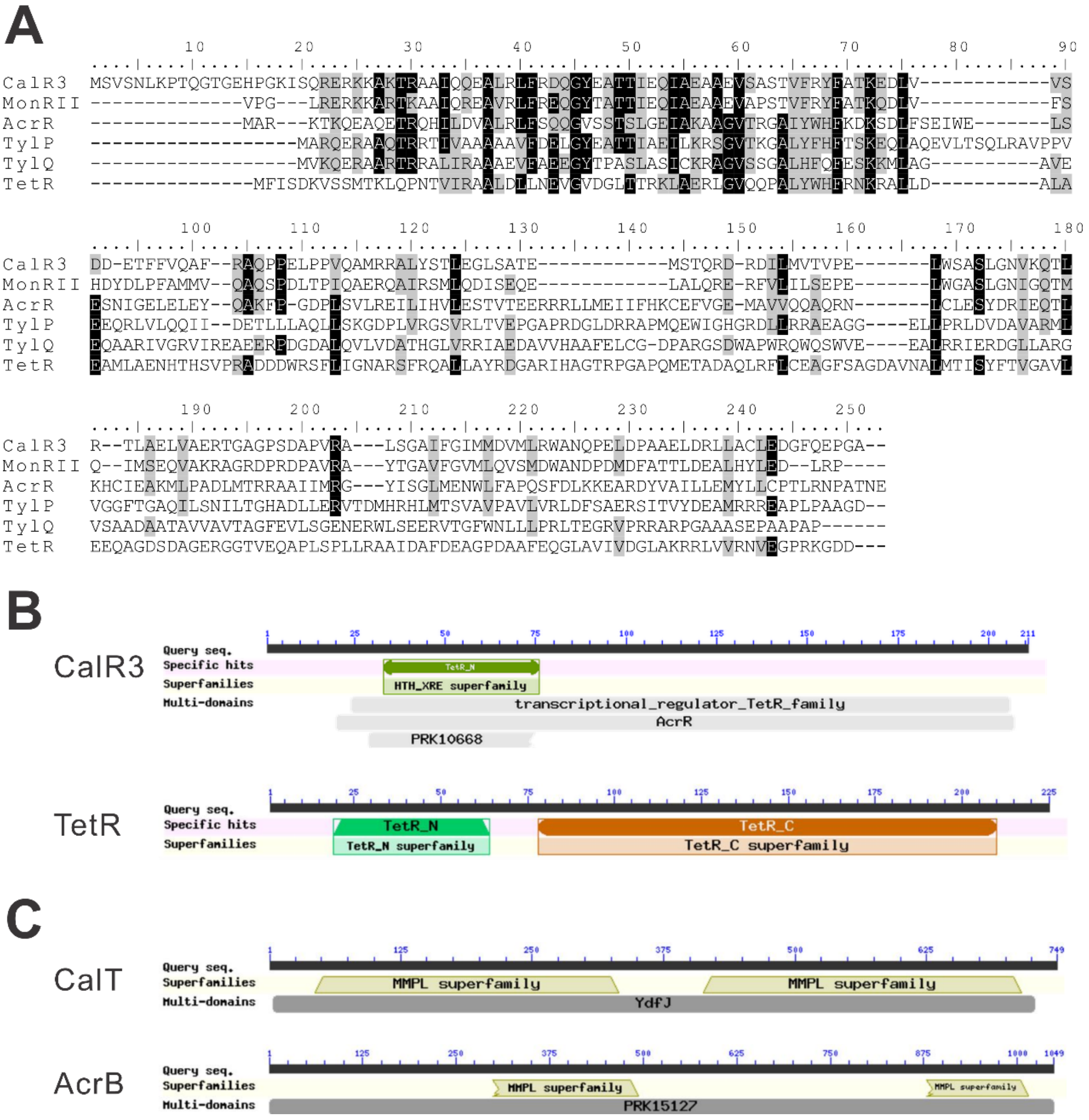

Supplementary Figure 1. *In silico* analysis of CalR3 and CalT.

A, Amino acid sequence alignment of CalR3 and its homologs.

B, Conserved functional domain prediction of CalR3 and its comparison with TetR.

C, Conserved functional domain prediction of CalT and its comparison with AcrB, a multidrug efflux protein in *E. coli*.

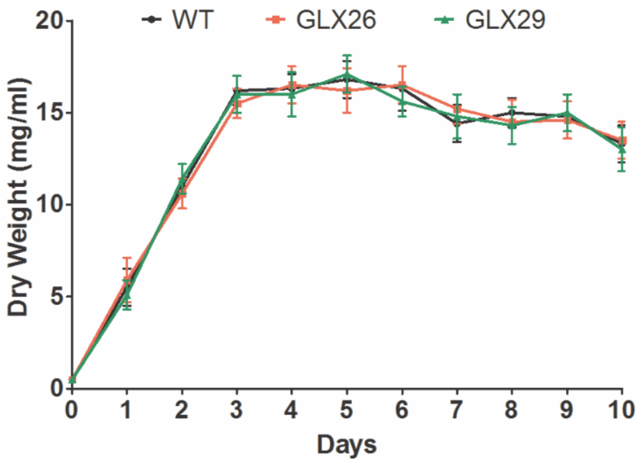

Supplementary Figure 2. Growth curves of the WT, GLX26(*ΔcalR3*) and GLX29 (*ΔcalR3:calR3*) strains. Cell growth was measured as cell dry weight.

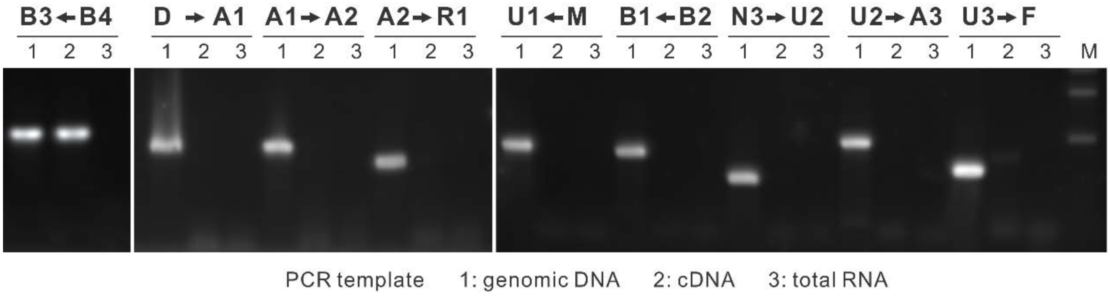

Supplementary Figure 3. Polycistron determination of *cal* genes.

The agarose gels show PCR fragments amplified from NRRL 3882 cDNA.

Amplification regions are presented as B3<-B4 etc. For example, B3 to B4 means the detected region between *calB3* and *calB4*, of which the arrows indicate the putative orientation of the transcription, and the corresponding amplifying primers were B3/B4-F1 and B3/B4-F2 (Table S1).

1, positive control using genomic DNA as the template; 2, cDNA as the template; 3, negative control using total RNA sample digested with DNase I as the template.

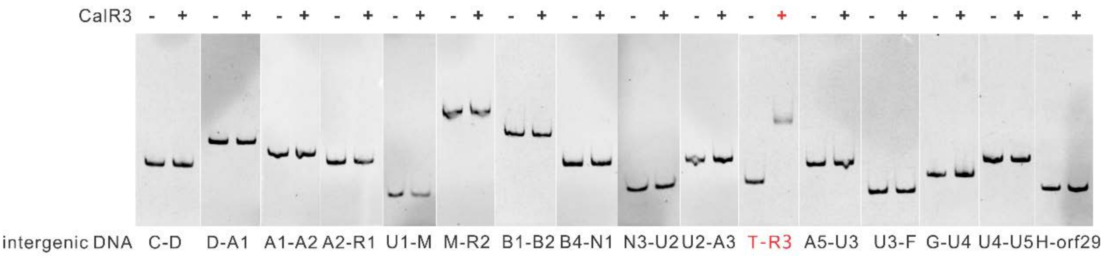

Supplementary Figure 4. EMSAs of the interaction of the His$_6$-CalR3 protein with the intergenic regions among *cal* genes.

C to D means the detected region between *calC* and *calD*. Lanes －, control reaction with inactivated protein; lanes +, EMSA reaction with the His$_6$-CalR3 protein.

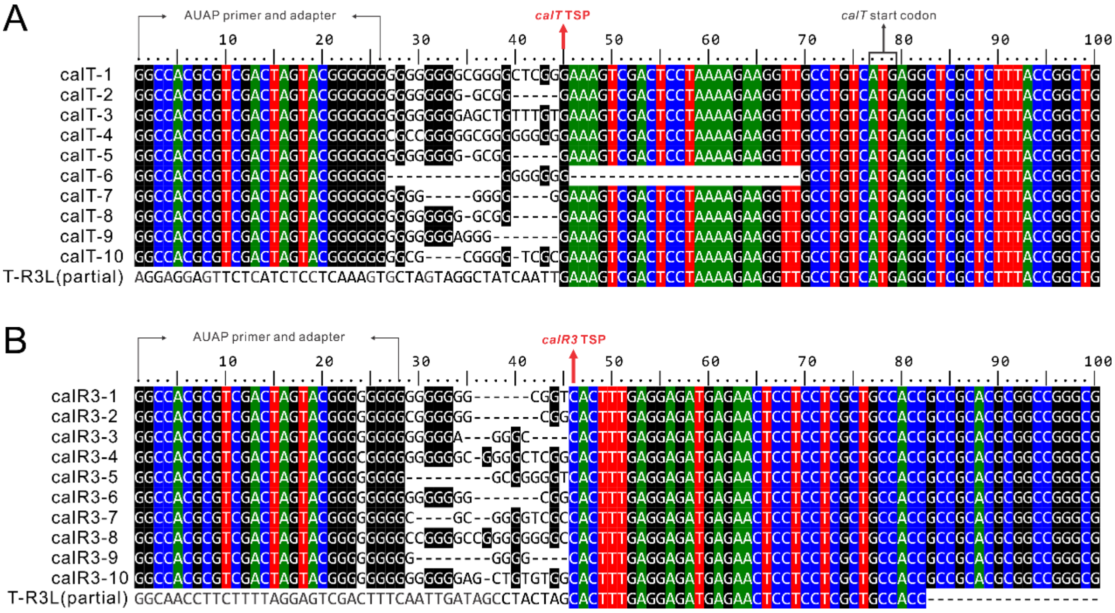

Supplementary Figure 5. 5'RACE sequencing and alignment results of *calT* (panel A) and *calR3* (panel B).

The region covered by the bent arrow represents the AUAP amplifying primer and adapters.

For each gene, ten clones were tested and analyzed. The precise TSP is denoted by a red arrow.

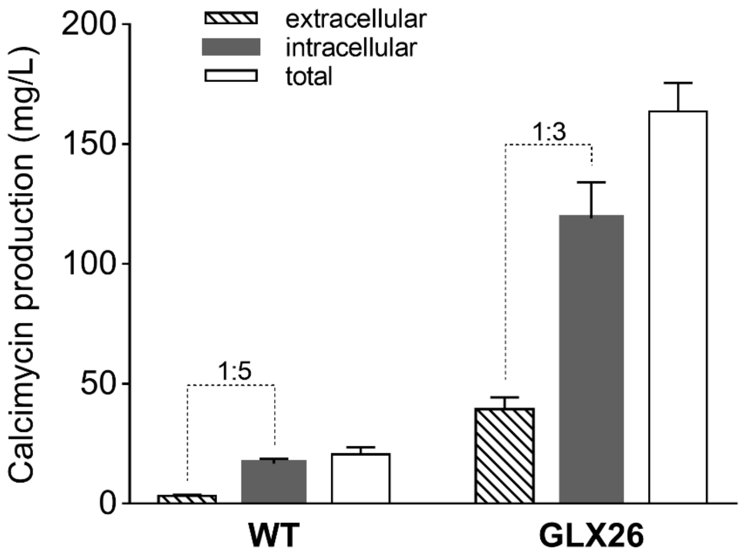

Supplementary Figure 6. Extracellular and intracellular calcimycin production in WT and GLX26 strains.

The “total” group is measured individually as a parallel control to assess the total calcimycin production of WT and GLX26 strains.
